# SMAD1 Loss-of-Function Variant Responsible for Congenital Heart Disease

**DOI:** 10.1155/2022/9916325

**Published:** 2022-03-03

**Authors:** Zhi Wang, Xiao-Hui Qiao, Ying-Jia Xu, Xing-Yuan Liu, Ri-Tai Huang, Song Xue, Hai-Yan Qiu, Yi-Qing Yang

**Affiliations:** ^1^Department of Pediatric Internal Medicine, Ningbo Women & Children's Hospital, Ningbo 315031, China; ^2^Department of Cardiology, Shanghai Fifth People's Hospital, Fudan University, Shanghai 200240, China; ^3^Department of Pediatrics, Tongji Hospital, Tongji University School of Medicine, Shanghai 200065, China; ^4^Department of Cardiovascular Surgery, Renji Hospital, School of Medicine, Shanghai Jiao Tong University, Shanghai 200127, China; ^5^Department of Cardiovascular Research Laboratory, Shanghai Fifth People's Hospital, Fudan University, Shanghai 200240, China; ^6^Department of Central Laboratory, Shanghai Fifth People's Hospital, Fudan University, Shanghai 200240, China

## Abstract

As the most common form of developmental malformation affecting the heart and endothoracic great vessels, congenital heart disease (CHD) confers substantial morbidity and mortality as well as socioeconomic burden on humans globally. Aggregating convincing evidence highlights the genetic origin of CHD, and damaging variations in over 100 genes have been implicated with CHD. Nevertheless, the genetic basis underpinning CHD remains largely elusive. In this study, via whole-exosome sequencing analysis of a four-generation family inflicted with autosomal-dominant CHD, a heterozygous *SMAD1* variation, NM_005900.3: c.264C > A; p.(Tyr88∗), was detected and validated by Sanger sequencing analysis to be in cosegregation with CHD in the whole family. The truncating variation was not observed in 362 unrelated healthy volunteers employed as control persons. Dual-luciferase reporter gene assay in cultured COS7 cells demonstrated that Tyr88∗-mutant SMAD1 failed to transactivate the genes *TBX20* and *NKX2.5*, two already well-established CHD-causative genes. Additionally, the variation nullified the synergistic transcriptional activation between SMAD1 and MYOCD, another recognized CHD-causative gene. These data indicate *SMAD1* as a new gene responsible for CHD, which provides new insight into the genetic mechanism underlying CHD, suggesting certain significance for genetic risk assessment and precise antenatal prevention of the family members inflicted with CHD.

## 1. Introduction

Congenital heart disease (CHD) constitutes the most frequent type of birth deformity in humans, inflicting ~1% of live births globally [1, 2]. Based on cardiac anatomic abnormalities, CHD is clinically categorized into >20 distinct types, including pulmonary stenosis (PS), ventricular septal defect (VSD), atrial septal defect, patent ductus arteriosus (PDA), and tetralogy of Fallot [1]. CHD may result in neurodevelopmental abnormality, thromboembolic complications, infective endocarditis, pulmonary hypertension, heart failure, arrhythmias, and death [3, 4]. Tremendous advancement has been achieved in pediatric cardiovascular surgery during recent decades, which enables the overwhelming majority (up to 97%) of neonates with CHD to survive childhood and reach adulthood, and now, CHD adults outnumber CHD children [5, 6]. Unfortunately, prolonged life span has led to an increasing number of adult CHD survivors, who are prone to suffering from miscellaneous late complications, with heart failure and cardiac arrhythmias being the most prominent [7, 8]. Consequently, CHD has conferred substantial morbidity and mortality as well as socioeconomic burden on humans [1]. Despite clinical importance, the etiologies accountable for CHD remain largely obscure.

It has been demonstrated that cardiac morphogenesis is a sophisticated biological process and both genetic defects and nongenetic precipitating risk factors may disturb this complex process, giving rise to CHD [2, 9–14]. Well-documented nongenetic risk factors predisposing to CHD include maternal diabetes, folate deficiency, viral infections, autoimmune disorder, and environmental exposures to air pollutants and medications [9]. However, aggregating evidence indicates that genetic components exert a key effect on the occurrence of CHD, and in addition to chromosomal alterations (aneuploidies) and copy number variations, pathogenic mutations in >100 genes, including *TBX20*, *NKX2.5*, and *MYOCD*, have been involved in the occurrence of CHD [2, 10–14]. Nevertheless, the genetic culprit components for CHD in up to 55% of cases remain unveiled [12]. Hence, there is still much research work to be fulfilled to show a complete picture of genetic causes for CHD.

## 2. Materials and Methods

### 2.1. Recruitment of Research Participants

The current research was completed in conformity with the guidelines of the World Medical Association Declaration of Helsinki. Approval of the research protocol was achieved from the local institutional medical ethics committee, with an ethical approval number of LL(H)-09-07. Written informed consent was provided by the study participants or their parents. For this research, a four-generation pedigree with high incidence of autosomal-dominant CHD was enlisted. A total of 362 unrelated ethnicity-matched volunteers without CHD were recruited as control individuals. All research participants experienced a comprehensive clinical investigation, encompassing review of personal and medical histories as well as familial histories, careful physical examination, transthoracic echocardiogram, and electrocardiogram. Diagnosis of CHD was made as previously described [15]. Peripheral blood specimen was collected from each study subject, and genomic DNA was prepared from blood leucocytes of each study subject.

### 2.2. Molecular Genetic Studies

For a study participant, a whole-exome library was prepared using 2 *μ*g of genomic DNA and captured with the SureSelect Human All Exon V6 Kit (Agilent Technologies), as per the manufacturer's manual. The exome library was enriched and then sequenced on the Illumina HiSeq 2000 Genome Analyzer (Illumina) using the HiSeq Sequencing Kit (Illumina), following the protocol. Bioinformatics assays of the data produced by whole-exome sequencing (WES) were performed as previously described [16–19]. The candidate variants identified by WES and bioinformatical analyses of the DNA samples from the CHD family underwent Sanger sequencing analysis in the whole family with CHD. For a verified genetic variation, the entire coding region and splicing donors/acceptors of the gene were sequenced in all available family members of the family with CHD and 362 unrelated control persons and such population genetics database as the Single Nucleotide Polymorphism (SNP) Database (https://www.ncbi.nlm.nih.gov/) and the Genome Aggregation Database (gnomAD; https://gnomad.broadinstitute.org/) were consulted to check its novelty.

### 2.3. Generation of Eukaryotic Gene Expression Plasmids

Preparation of cDNAs from discarded human heart samples was described elsewhere [4]. The full-length cDNA of wild-type human *SMAD1* (accession no. NM_005900.3) was amplified on a thermocycler (Applied Biosystems) by polymerase chain reaction (PCR) utilizing high-fidelity DNA polymerase (Stratagene) with a specific pair of primers (forward: 5′-GACGCTAGCCCAAGGAGTATAACTAGTGC-3′; backward: 5′-GTCCTCGAGGTCTGACTCATCCATCCTTC-3′). The produced *SMAD1* cDNAs and the pcDNA3.1 plasmid DNAs were doubly digested by *Nhe*I (NEB) and *Xho*I (NEB), respectively and then purified with a gel extraction kit (Qiagen), and finally, the purified *SMAD1* cDNA was inserted into the pcDNA3.1 plasmid at the *Nhe*I-*Xho*I sites to create a recombinant SMAD1-pcDNA3.1 expression plasmid. The identified genetic variation was introduced into wild-type SMAD1-pcDNA3.1 via site-directed mutagenesis using a site-directed mutagenesis kit (Stratagene) and a complementary pair of primers (forward: 5′-GCCTCATGTCATTTAATGCCGTGTGTGGCGC-3′; backward: 5′-GCGCCACACACGGCATTAAATGACATGAGGC-3′) and was validated by Sanger sequencing. Likewise, the full-length cDNA of wild-type human *MYOCD* (accession no. NM_001146312.3) was amplified by PCR, doubly digested with *Nhe*I (NEB) and *Xho*I (NEB), and subcloned into pcDNA3.1 (doubly digested with *Nhe*I and *Xho*I) at the *Nhe*I-*Xho*I sites to create the eukaryotic expression plasmid MYOCD-pcDNA3.1. A 980 bp fragment (from –980 to –1) of human *TBX20* (accession no. NC_000007.14) was amplified by PCR from human genomic DNA utilizing high-fidelity DNA polymerase (Stratagene) and a specific pair of primers (forward: 5′-GTTGCTAGCGTCAGCCTGAGTTTACACGG-3′; backward: 5′-AACCTCGAGCCTGGCGCTCGCTGCCCTGC-3′), cut with *Nhe*I (NEB) and *Xho*I (NEB), and inserted into the promoter-less pGL3-basic vector (Promega), which was cut with *Nhe*I (NEB) and *Xho*I (NEB), to generate a *TBX20* promoter-driven firefly luciferase reporter vector (TBX20-luc). Similar with a previous report [20], a human *NKX2.5* promoter-driven firefly luciferase reporter vector (NKX2.5-luc) was constructed. Each construct was confirmed by Sanger sequencing analysis.

### 2.4. Cellular Transient Transfection and Reporter Gene Analysis

COS7 cells were maintained in Dulbecco's modified Eagle's medium (Thermo Fisher Scientific) with 10% fetal bovine serum (Gibco), 100 IU/mL penicillin (Sigma-Aldrich,), and 100 *μ*g/mL streptomycin (Sigma-Aldrich), in a cell culture incubator at 37°C with an atmosphere of 95% air as well as 5% CO_2_. Cells were cultured in a 12-well plate 24 h before transient transfection. Cells were transiently transfected with expression plasmids by employing the ViaFect™ Transfection Reagent (Promega). The plasmid pGL4.75 (Promega), which expresses Renilla luciferase, was cotransfected as an internal control to balance transfection efficiency, and the total amount of various plasmid DNAs per well was kept constant by supplementing the empty plasmid pcDNA3.1 where necessary. Unless otherwise indicated, 1000 ng of firefly luciferase reporter plasmid (TBX20-luc or NKX2.5-luc), 20 ng of Renilla luciferase control plasmid (pGL4.75), and 200 ng of each activator expression plasmid (wild-type SMAD1-pcDNA3.1, Tyr88∗-mutant SMAD1-pcDNA3.1 or MYOCD-pcDNA3.1, singly or in combination) were used. The luciferase activities were analyzed as described previously [21], with a dual-luciferase analysis system (Promega).

### 2.5. Statistical Analysis

The activity of a promoter was given as a ratio of firefly luciferase to Renilla luciferase. Values for promoter activity were expressed as mean ± standard deviation (SD) of the results from three transfection experiments in triplicate. Student's *t*-test was applied to statistical analysis. A two-sided *p* < 0.05 denoted statistical difference.

## 3. Results

### 3.1. Clinical Characteristic Information of the Study Pedigree

**Figure 1:**
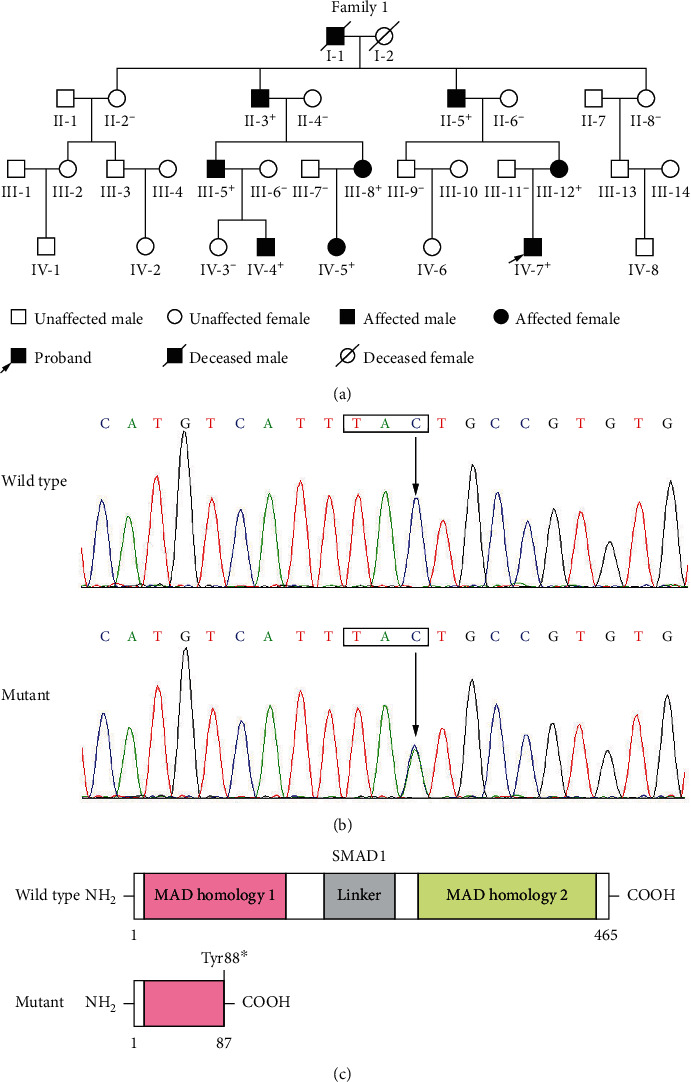
Novel *SMAD1* mutation underpinning familial congenital cardiac deformity. (a) Pedigree suffering from congenital heart deformities. Pedigree members are identified by generations and numbers. “^+^” Represents a carrier of the heterogeneous *SMAD1* variation; “^–^” means a noncarrier. (b) Sequence chromatogram traces illustrating the *SMAD1* variation (in heterozygous status) from the index patient (mutant) and its wild-type control (in homozygous status) from a healthy family member (wild type). A rectangle symbol marks a genetic codon, with an arrow directing the nucleotide site where the mutation occurred. (c) Schemas describing the functional domains of SMAD1. NH2: amino-terminus; MAD: mothers against decapentaplegic; COOH: carboxyl-terminus.

In the present investigation, a large family with CHD spanning four generations (Figure 1(a)) was enrolled from the Chinese Han-race population. In this Chinese family, there were 32 family members, including 30 living members (15 male members and 15 female members, with ages ranging from 3 to 55 years) and all the nine affected members had echocardiogram-documented PDA. In addition, four members also suffered from VSD and three members also suffered from PS. Genetic analysis of this four-generation pedigree (Figure 1(a)) revealed that PDA was inherited in an autosomal-dominant fashion, with 100% penetrance. The proband, a three-year-old boy, underwent catheter-based cardiac repairment due to PDA and VSD. The proband's affected relatives also underwent interventional procedures for correction of CHD, except for his mother's grandfather (I-1), who died of congestive heart failure at the age of 69 years. No recognized noninherited risk factors contributing to CHD were ascertained in all the family members. The basic clinical information of the affected family members with CHD is provided in Table 1.

### 3.2. Detection of a CHD-Causing SMAD1 Mutation

WES was carried out in four CHD-inflicted family members (including III-5, III-12, IV-4, and IV-7) and three unaffected family members (including III-6, III-11, and IV-3) of the family (Figure 1(a)), yielding approximately 22 Gb of sequence data for each study family member, with ~98% locating to the human genome (hg19) and~75% mapping to the target sequences. A mean of 16,206 exonic variants (ranging from 15,382 to 17,098) per study family member that passed filtering by the inheritance model, of which 11 heterozygous nonsense and missense variants passed filtering by ANNOVAR, was carried by the four CHD-inflicted family members and was predicted to be disease causing, with a minor allele frequency < 0.1%, as shown in Table 2. Further genetic assays unveiled that only the variant chr4:146,436,029C > A (GRCh37: NC_000004.11), equivalent to chr4:145,514,877C > A (GRCh38: NC_000004.12) or NM_005900.3: c.264C > A; p.(Tyr88∗), in *SMAD1*, was verified by Sanger sequencing with the primer pairs given in Table 3 and was shown to be in cosegregation with CHD in the entire family. The electropherogram traces illustrating the *SMAD1* variation in the heterozygous status as well as its wild-type control base are presented in Figure 1(b). The schemas exhibiting the key functional domains of wild-type SMAD1 and Tyr88∗-mutant SMAD1 are exhibited in Figure 1(c). The identified *SMAD1* variant was neither observed in 724 control chromosomes nor reported in the databases of SNP and gnomAD, suggesting a novel *SMAD1* variant. This *SMAD1* variant, NM_005900.3: c.264C > A; p.(Tyr88∗), was submitted to Leiden Open Variation Database (LOVD; https://databases.lovd.nl/shared/genes/SMAD1), with a variant number of 0000838399 (https://databases.lovd.nl/shared/variants/0000838399#00019404) and an individual identity number of 00401998 (https://databases.lovd.nl/shared/individuals/00401998).

### 3.3. Functional Loss of Tyr88∗-Mutant SMAD1

**Figure 2:**
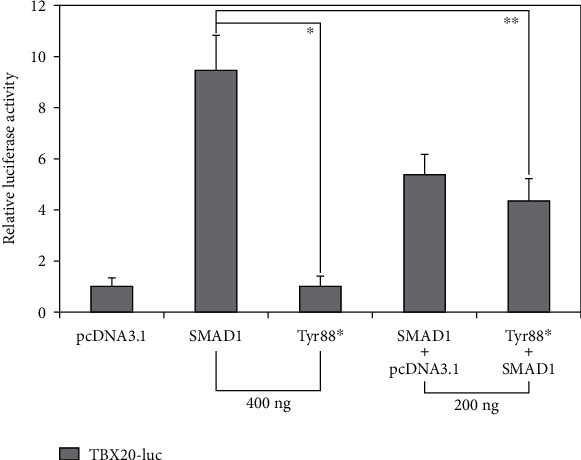
Functional impairment of SMAD1 caused by the variation. Dual-luciferase reporter analysis of the transcriptional activation of *TBX20* promoter-driven firefly luciferase in cultured COS7 cells by equal amount of wild-type or Tyr88∗-mutant SMAD1 plasmid, singly or in combination, demonstrated that Tyr88∗-mutant SMAD1 lost transcriptional activity. Three transient transfection experiments were performed in triplicate for every expression plasmid. The results are reported as the mean ± standard deviation of the data from three independent reporter assays. ^∗^*p* < 0.001 and ^∗∗^*p* < 0.01, when compared with wild-type SMAD1 (400 ng).

As shown in Figure 2, wild-type SMAD1 and Tyr88∗-mutant SMAD1 transactivated the promoter of *TBX20* by ~9-fold and ~1-fold, respectively (wild-type SMAD1 versus Tyr88∗-mutant SMAD1: *t* = 10.0207; *p* = 0.00056). When wild-type SMAD1 and Tyr88∗-mutant SMAD1 was cotransfected, the induced transcriptional activity was ~4-fold (wild-type SMAD1 + empty plasmid pcDNA3.1 versus wild-type SMAD1 + Tyr88∗-mutant SMAD1: *t* = 5.4032; *p* = 0.00568).

### 3.4. Nullified Synergistic Transcriptional Activation Between SMAD1 and MYCOD by the Tyr88∗ Mutation

**Figure 3:**
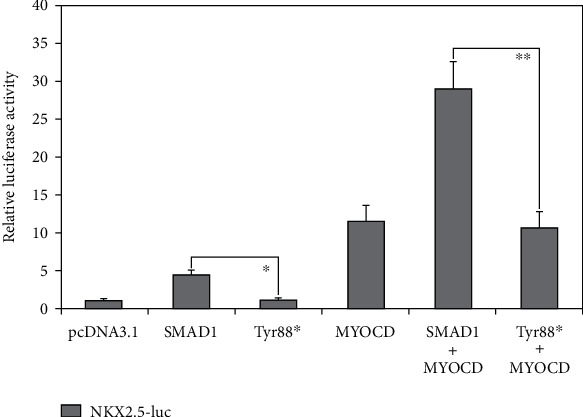
Nullified synergistic transactivation between SMAD1 and MYOCD by the variation. Biological measurements of the synergistic transcriptional activation of the NKX2.5 promoter in cultivated COS7 cells by SMAD1; MYOCD was abrogated by the Tyr88∗ variation. Three cellular transfection experiments were repeated in triplicate for every expression plasmid. All values are given as the mean ± standard deviation of the results from three independent reporter gene analyses. Both ^∗^ and ^∗∗^ indicate that *p* < 0.005, in comparison with their corresponding wild-type counterparts.

As shown in Figure 3, wild-type SMAD1 and Tyr88∗-mutant SMAD1 transactivated the promoter of *NKX2.5* by ~4-fold and ~1-fold, respectively (wild-type SMAD1 versus Tyr88∗-mutant SMAD1: *t* = 8.08764; *p* = 0.00127). In the presence of wild-type MYCOD, wild-type SMAD1 and Tyr88∗-mutant SMAD1 transcriptionally activated the promoter of *NKX2.5* by ~29-fold and ~11-fold, respectively (wild-type SMAD1 + wild-type MYCOD versus Tyr88∗-mutant SMAD1 + wild-type MYCOD: *t* = 7.33082; *p* = 0.00184).

## 4. Discussion

In the present study, a four-generation Chinese family suffering from CHD transmitted as an autosomal dominant trait was enrolled. Via WES analysis of the DNA samples from the family members, a novel variation in the *SMAD1* gene, NM_005900.3: c.264C > A; p.(Tyr88∗), was identified, which was confirmed by Sanger sequencing analysis to be in cosegregation with CHD in the whole family. The heterozygous variation was absent from 724 control chromosomes nor reported in the databases of SNP and gnomAD. Reporter gene assays demonstrated that Tyr88∗-mutant SMAD1 failed to transcriptionally activate the promoters of *TBX20* and *NKX2.5*, two well-established CHD-causing genes [22, 23]. Moreover, the mutation nullified the synergistic transcriptional activation between SMAD1 and MYOCD, another CHD-causative gene [11, 24–26]. These observational results indicate that genetically compromised *SMAD1* predisposes to CHD.

*SMAD1* maps on human chromosome 4q31.21, coding for a protein comprising 465 amino acids, a member of the SMAD superfamily of proteins like the products of the *Sma* gene from *Caenorhabditis elegans* and the *Mad (Mothers against decapentaplegic)* gene from *Drosophila* [27, 28]. As a transcription factor and signal transducer that regulates multiple signal pathways, the SMAD1 protein possesses two evolutionarily conserved structural domains, MAD homology 1 (MH1) and MAD homology 2 (MH2), which are separated by Linker [29]. MH1 functions mainly to bind to the DNA consensus sequence of GNCN in target gene promoters and to transcriptionally activate the expression of target genes, in addition to mediating nuclear accumulation of SMAD1 and interaction of SMAD1 with other transcription factors. MH2 is responsible for the interaction of SMAD1 with a wide variety of proteins, provides selectivity and specificity to SMAD1 function, contributes to the binding affinity, and is involved in nuclear accumulation of SMAD1. Linker connecting MH1 and MH2 contains multiple critical peptide motifs, encompassing a nuclear export signal and several potential phosphorylation sites, and hence has a role in transcriptional activation [29]. SMAD1 is highly expressed in the heart throughout embryogenesis, playing a crucial role in transactivating the expression of target genes essential for cardiovascular morphogenesis, including *TBX20*, *NKX2.5*, *ACTC1*, and *MYH6*, alone or synergistically with MYOCD and TBX20 [30–32]. Furthermore, loss-of-function variations in *TBX20*, *NKX2.5*, *ACTC1*, *MYH6*, and *MYOCD* have been involved in the molecular pathogenesis of CHD [11, 22, 23, 33–36]. In the current study, the variation discovered in cases with familial CHD was predicted to generate a truncating SMAD1 protein lacking MH2 and Linker as well as a part of MH1 and functional data demonstrated that Tyr88∗-mutant SMAD1 failed to transactivate its downstream target genes. These findings support that haploinsufficiency of *SMAD1* is the genetical mechanism of CHD which occurred in this family.

It may be ascribed to abnormal cardiovascular development that *SMAD1* mutation gives rise to CHD. In most animal species, including xenopus, zebrafish, mice, rats, and humans, SMAD1 is amply expressed in the cardiovascular system during embryonic development, playing a pivotal role in cardiovascular morphogenesis via mediating the cellular proliferation, growth, apoptosis, differentiation, and morphogenesis in the heart and vessels [29, 37]. In mice, homozygous knockout of *Smad1* led to embryonic demise because the embryos failed to seed the placenta and the *Smad1*-null embryos showed markedly impaired allantois formation and drastically reduced primordial germ cells; while the embryos with heterozygous deletion of *Smad1* developed remarkably normally, probably due to functional compensation by *Smad5* and *Smad8*, which shared common expression profiles and functional characteristics with *Smad1* [38, 39]. Although the mice with heterozygous knockout of either *Smad1* (*Smad1*^+/−^) or *Smad5* (*Smad5*^+/−^) developed properly, the murine embryos with double heterozygous knockout of *Smad1* and *Smad5* (*Smad1*^+/−^/*Smad5*^+/−^) died by E10.5 and the double heterozygous embryos (*Smad1*^+/−^/*Smad5*^+/−^) presented defects of heart looping and laterality [39]. Furthermore, the mice with conditional knockout of the *Smad1* gene by disrupting *Smad1* either in endothelial cells or in smooth muscle cells displayed increased pulmonary pressure, right ventricular hypertrophy, and thickened pulmonary arterioles [40]. Collectively, these results from experimental animals suggest that genetically compromised *SMAD1* predisposes to CHD in human beings.

## 5. Conclusions

The current investigation indicates *SMAD1* as a novel causative gene responsible for CHD, which provides a new potential target for antenatal prophylaxis and personalized treatment of CHD patients.

## Figures and Tables

**Table 1:** Clinical characteristic data and *SMAD1* variation status of the pedigree members suffering from congenital heart disease.

Individual (family 1)	Gender	Age (years)	Cardiac structural defects	SMAD1 variation (Tyr88∗)
I-1	Male	69^∗^	PDA, VSD, PS	NA
II-3	Male	55	PDA	+/–
II-5	Male	52	PDA, VSD, PS	+/–
III-5	Male	29	PDA	+/–
III-8	Female	27	PDA	+/–
III-12	Female	26	PDA, VSD, PS	+/–
IV-4	Male	6	PDA	+/–
IV-5	Female	4	PDA	+/–
IV-7	Male	3	PDA, VSD	+/–

NA: not available; PDA: patent ductus arteriosus; PS: pulmonary arterial stenosis; VSD: ventricular septal defect; +/–: heterozygote for the SMAD1 variation. ^∗^Age at death.

**Table 2:** Nonsynonymous variations in candidate genes for congenital heart disease discovered via whole-exome sequencing as well as bioinformatical analysis.

Chr	Position (GRCh37)	Ref	Alt	Gene	Variation
1	44,595,817	A	C	KLF17	NM_173484.4: c.874A>C; p.(Lys292Gln)
1	223,176,637	T	G	DISP1	NM_032890.5: c.1898T>G; p.(Phe633Cys)
2	180,383,314	C	A	ZNF385B	NM_152520.6: c.448C>A; p.(Pro150Thr)
3	118,913,103	A	T	UPK1B	NM_006952.4: c.506A>T; p.(Gln169Leu)
4	146,436,029	C	A	SMAD1	NM_005900.3: c.264TC>A; p.(Tyr88∗)
5	10,649,993	G	A	ANKRD33B	NM_001164440.2: c.1253G>A
7	147,092,783	C	G		; p.(Arg418Gln)
11	40,137,545	A	G	CNTNAP2	NM_014141.6: c.1581C>G; p.(Asp527Glu)
14	34,263,135	T	C	LRRC4C	NM_020929.3: c.298A>G; p.(Arg100Gly)
16	72,993,460	C	G	NPAS3	NM_001164749.2: c.1186T>C;
20	58,416,533	G	T	ZFHX3	p.(Tyr396His)
				PHACTR3	NM_006885.4: c.585C>G; p.(Ile195Met)
					NM_080672.5: c.1530G>T; p.(Ala510Ser)

Alt: alteration; Chr: chromosome; Ref: reference.

**Table 3:** Primers for amplification of the coding exons as well as splicing junctions of the *SMAD1* gene.

Coding exons	Forward primers (5′ → 3′)	Backward primers (5′ → 3′)	Amplicons (bp)
1	TGTCCTTTTGCATTTGGAGAC	CAAATCTGGTACTGGGCACAC	518
2	TTGAGTTGGCAGCAGGACAG	ACTGCAGGTTGACCCAGCTT	473
3	GGCAGTGCCTGTAGCCTTTAG	CCAGCAATTGTAGTGAGCTTCT	412
4	CCATGATCCTGAGCCAATTC	C	483
5	CTGTGTTGAAGCTGCACAGG	TGCAAGAGCTTCCAGATAGCAG	590
6	CAGGGAGGAAAGATGCATAG	AGCGGTGCTATCTGAATAAGGA	569
	C	ACAATTTGTCCCTGGCTTGG	

## Data Availability

The data supporting the findings of this investigation are available upon reasonable request.
